# A Prognostics Method Based on Back Propagation Neural Network for Corroded Pipelines

**DOI:** 10.3390/mi12121568

**Published:** 2021-12-16

**Authors:** Mingjiang Xie, Zishuo Li, Jianli Zhao, Xianjun Pei

**Affiliations:** School of Mechanical Engineering, Southeast University, Nanjing 211189, China; mingjiang@seu.edu.cn (M.X.); LiZishuo@sjtu.edu.cn (Z.L.); 220210314@seu.edu.cn (J.Z.)

**Keywords:** pipeline corrosion, BP neural network, uncertainty, corrosion growth model

## Abstract

A method that employs the back propagation (BP) neural network is used to predict the growth of corrosion defect in pipelines. This method considers more diversified parameters that affect the pipeline’s corrosion rate, including pipe parameters, service life, corrosion type, corrosion location, corrosion direction, and corrosion amount in a three-dimensional direction. The initial corrosion time is also considered, and, on this basis, the uncertainties of the initial corrosion time and the corrosion size are added to the BP neural network model. In this paper, three kinds of pipeline corrosion growth models are constructed: the traditional corrosion model, the corrosion model considering the uncertainties of initial corrosion time and corrosion depth, and corrosion model also considering the uncertainties of corrosion size (length, width, depth). The rationality and effectiveness of the proposed prediction models are verified by three case studies: the uniform model, the exponential model, and the gamma process model. The proposed models can be widely used in the prediction and management of pipeline corrosion.

## 1. Introduction

With the global economy and industrialization developing rapidly, the demand for oil and natural gas gradually increases. The primary transportation method for the above two resources is pipeline transportation. However, because of the long-distance transportation of oil and gas pipelines, natural corrosion, third party damage, and other reasons, the pipeline’s wall thickness will attenuate, thus affecting the service life of the pipelines [1,2,3,4]. Among many failure types, pipeline failure caused by corrosion defects accounts for a large proportion. Many pipelines have been in service for more than ten years, and some of their structures are seriously corroded. Pipeline accidents caused by corrosion defects occur from time to time, becoming a significant threat of the pipeline. These pipeline failures could potentially pollute the environment, waste energy, and threaten public lives and property safety.

To ensure the integrity of corroded pipelines, it is necessary to take corresponding measures to predict the remaining useful life (RUL) of the pipelines [3,5,6]. With the exploration and research of many scientific experts, pipeline corrosion detection and life prediction have been studied a lot. In recent years, breakthroughs have been made in data acquisition, machine learning, and other fields, providing new theoretical support and prognostics methods for the degradation of corrosion defects in pipelines. Continuously improving the accuracy of pipeline corrosion depth prediction and RUL prediction can provide additional benefits to the arrangement of inspection and maintenance actions of pipelines, and further reduce life-cycle costs.

Inline inspection (ILI) tools are widely used to detect and inspect the location and size of pipeline corrosion defects [7,8,9,10]. The accuracy of the ILI tool has a great impact on the prediction results. Simple and improved Monte Carlo simulations (MCS) [11,12,13] are used to calculate the failure probability of a section of corroded pipeline considering the uncertainty of corrosion process. The first-order reliability method (FORM) is also used to evaluate the reliability of pipelines by linearizing the relationship between reliability and parameters of pipelines [14,15]. With the increasing number of variables, MCS and FORM methods can be relatively time-consuming. Aiming at the complex nonlinear relationship between pipeline parameters and corrosion, Ozan [16] utilized artificial neural networks (ANNs) to predict the remaining useful life. Tian [17] took the pipeline’s service life and state detection value as the input of the neural network and the life percentage as the output. The neural network model has the key advantage of dealing with nonlinear relationship between pipeline parameters and corrosion growth.

Back propagation (BP) neural network is one kind of artificial neural network that has high prediction accuracy and has been applied to predict the life of pipelines [18,19]. In this paper, a method based on BP neural network is used to simulate and predict corrosion defect growth. The related application of this method has been studied in some literature. Kai et al. [20] used the artificial neural network method to simulate the growth of corrosion defect, and evaluated the structural safety and reliability of pipeline. However, to simplify the structure of the neural networks, they only consider the internal pressure of the corroded pipeline. To assess the risk of the pipeline, Raeihagh et al. [21] established a fuzzy inference system (FIS), and applied the selected factors to the artificial neural networks (ANNs). Ben et al. [22] applied six artificial intelligence models, such as ANN, multivariate adaptive regression splines (MARS), and M5 tree (M5Tree) to study the relationship between the depth of corrosion and probable factors. These studies rarely consider the uncertainty of corrosion size and mostly ignore the initial corrosion time of the pipeline, which may produce inaccurate estimations. What is more, different manufacturing processes at different positions of the pipeline will also affect the corrosion growth of the pipeline. For example, high-speed particles will impact welded nodes [23], which have a relatively high risk. Similarly, only a few pieces of literature have made research on these factors. In this paper, we consider more diversified corrosion parameters, including pipe parameters, corrosion type, service life, corrosion location, corrosion direction, and corrosion size in a three-dimensional direction. In addition, considering the uncertainties of inspection data and initial corrosion time of pipeline, we build an ANN model for the degradation of corroded pipelines and consider the influence of other uncertainty sources to verify the effectiveness of the methodology.

The rest of the paper is organized as follows. Section 2 describes the structure, the modeling process, and the performance assessment of the BP neural network model. Section 3 presents data preprocessing and three prognostics models based on the BP neural network. In Section 4, three case studies with different corrosion growth models are used to demonstrate the effectiveness of the proposed models. Conclusions are presented in Section 5.

## 2. BP Neural Network Model

Since the relationship between input variables (including pipe properties, corrosion location, corrosion size, corrosion type, etc.) and the corrosion growth is very complex, finding a formula to describe the relationship is difficult. Considering that the BP neural network has strong ability to deal with nonlinear problems, as well as strong self-learning and self-adaptive abilities, a BP neural network is used to predict the corrosion growth of the pipeline. In this section, the structure, the modeling process, and the performance assessment of the BP neural network model will be described.

### 2.1. Structure of the BP Neural Network

**Figure 1:**
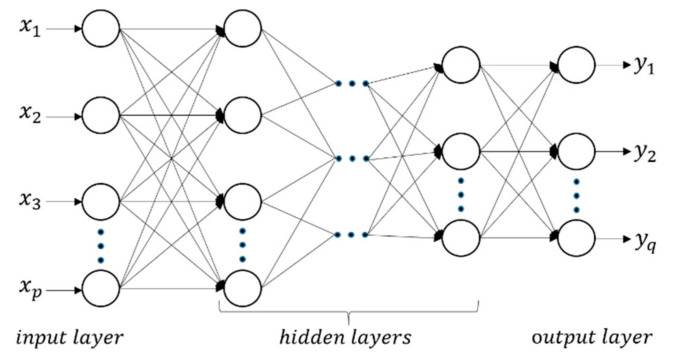
The structure of neural networks.

Being composed of many neurons with operation functions, the structure of the BP neural network includes the input layer, hidden layer, and output layer [19], which is shown in Figure 1. The input layer consists of *p* neurons represented by *x_i_*, *i* = 1, 2, 3, ..., *p*, where *p* is the number of input variables. The output layer consists of *q* neurons represented by *y_j_*, *j* = 1, 2, 3, ..., *q*, where *q* is the number of output variables. Each node of the input layer is connected to all the nodes of the first hidden layer. Each node of the previous hidden layer is connected with all the nodes of the next hidden layer. Similarly, each node of the last hidden layer is connected to the all the nodes of the output layer. Each connection has a weight associated with it. In this paper, 11 input variables (service life, pipe segment length, pipe wall thickness, corrosion type, corrosion location (distance to upstream/downstream girth weld, inner/outer, clock direction), and corrosion size (length, width, depth)) and 3 output variables (corrosion growth coefficients (length, width, depth)) are used to construct the BP neural network. Thus, the BP neural network has 11 input neurons and 3 output neurons, which means *p* = 11, *q* = 3.

The output of the neuron in the first hidden layer, $u_{k}^{1}$, *k* = 1, 2, … *H*_1_, where *H*_1_ is the number of neurons in the first hidden layer, is expressed as follows.

(1) $$p_{k}^{1}=\overset{p}{\underset{i=1}{\sum }}x_{i}z_{ik}^{1}$$

(2) $$u_{k}^{1}=f\left(p_{k}^{1}\right)$$

where *f* is a monotonically increasing function, whose value is within (0, 1). $z^{1}=\left[z_{11}^{1},\;z_{21}^{1},\;\ldots ,\;z_{p1}^{1},\;z_{12}^{1},\;z_{22}^{1},\;\ldots ,\;z_{p2}^{1},\;\ldots ,\;z_{1H_{1}}^{1},\;z_{2H_{1}}^{1},\;\ldots ,\;z_{pH_{1}}^{1}\right]$ is a vector of weights, and the initial values of these weights are all within [−1, 1]. Similarly, the output of the neuron in the *i*-th (*i* > 1) hidden layer, $u_{k}^{i}$, *k* = 1, 2, … *H_i_*, where *H_i_* is the number of neurons in the *i*-th hidden layer, is expressed as follows,

(3) $$p_{k}^{i}=\overset{H_{i-1}}{\underset{t=1}{\sum }}u_{t}^{i-1}z_{tk}^{i}$$

(4) $$u_{k}^{i}=f\left(p_{k}^{i}\right)$$

where $z^{i}=\left[z_{11}^{i},\;z_{21}^{i},\;\ldots ,\;z_{H_{i-1}1}^{i},\;z_{12}^{i},\;z_{22}^{i},\;\ldots ,\;z_{H_{i-1}2}^{i},\;\ldots ,\;z_{1H_{i}}^{i},\;z_{2H_{i}}^{i},\;\ldots ,\;z_{H_{i-1}H_{i}}^{i}\right]$ is also a vector of weights whose initial values are within [−1,1]. The output of the model *y_j_*, namely the output of the output neurons, is,

(5) $$l_{j}=\overset{H_{last}}{\underset{s=1}{\sum }}u_{s}^{H_{last}}v_{sj}$$

(6) $$y_{j}=f\left(l_{j}\right)$$

where $v=\left[v_{11},\;v_{21},\;\ldots ,\;v_{H_{last}1},\;v_{12},\;v_{22},\;\ldots ,\;v_{H_{last}2},\;\ldots ,\;v_{1q},\;v_{2q},\;\ldots ,\;v_{H_{last}q}\right]$ is a vector of weights within [−1,1], and *H_last_* is the number of neurons in the last hidden layer. In this paper, the sigmoid function is selected as the activation functions that can be used in (2), (4), and (6). This function is expressed as follows.

(7) $$f(x)=\frac{1}{1+e^{\left(-x\right)}}$$

The construction of a BP neural network is essentially the process of determining the weights of these connections. When a BP neural network works, it mainly transmits two kinds of data: the forward propagating signal and the back-propagating error. After the input data are obtained, its flow direction is taken from the input layer to the hidden layer, and then to the output layer. Then, the BP algorithm compares the actual outputs with the target outputs and the error is propagated in the opposite direction. The error is shared with each node of each layer, and the weight of each connection is adjusted until the objective function reaches the minimum value by using the back propagation learning rule. Then, the process of establishing the BP neural network is finished.

### 2.2. Modeling Process

The BP neural network is a data-driven model, and the modeling process is as follows:

- Obtain the database and determine the number of neurons in the input layer and output layer of the BP neural network;
- Randomly sort the collected data (data size = 11,103), and select = 70% of the samples (viz. 7772 data points) as the training samples. Then, the remaining samples are used as the testing samples;
- Train the BP neural network with the training samples and evaluate the performance of the model on the testing samples.

The BP model has two main limitations. The first one is the overfitting problem, which means the trained BP model has pretty high fitting precision on the training set, but has a relatively large prediction error on the testing set. In the proposed method, the target error of the BP model is not set too small, and the redundant samples are deleted. Then, this limitation is avoided. The second limitation is the inherent defect of the BP model and cannot be avoided. In the flat region of the gradient error surface, the variation of the weight is quite small, which makes the convergence of the BP model relatively slow. It spends more time in the training process.

In the process of establishing a BP neural network, the main content is to determine the neural network parameters, including the number of hidden layers, the number of nodes in each hidden layer, the learning rate, the learning objectives, and the frequency of training.

As for the determination of network parameters, we need to determine the number of hidden layers firstly. Then, we can get the number of nodes in each hidden layer according to the empirical formula, where $H_{i}$ represents the number of neurons in the *i*-th hidden layer.

(8) $$H_{i}=2\times i+3$$

In theory, the BP neural network of three hidden layers has a good fitting result. In this paper, using the collected data and a simple linear growth model, the BP neural network with one, two, three, four, five hidden layers are tested, respectively. The simulation result is the best when the BP neural network has four hidden layers. When the number of hidden layers is too large, such as five, the overfitting problem occurs. So, the number of hidden layers is set as four in this paper. Then, the number of neurons in these four hidden layers are 5, 7, 9, and 11.

Meanwhile, the other parameters of the BP neural network are proposed to use the default value [24]. The parameter selection of the BP neural network is shown in Table 1.

Based on the chosen parameters, the initial BP neural network model is built. After training the BP neural network with the training samples, the weights of the connections between neurons are optimized and the final BP neural network structure is determined. Then, the BP neural network is evaluated on the testing samples to verify its validity. After that, the established BP neural network can be used to predict the corrosion growth of the pipeline.

### 2.3. Performance Assessment

During the BP neural network training and testing process, a measure is needed to be determined to represent the applicability of the model. Expected value and variance are usually adopted in many papers. In this paper, using the corrosion depth of the pipeline in a year as the contrast quantity, we draw the predicted value ${\hat{x}}_{i}$ and the actual value $x_{i}$ on the same picture, and estimate the proximity between the predicted value and the expected actual value by analyzing the shape and trend of the curve. The mean square error (MSE) is selected to represent the performance of the model. The definition of MSE is as follows. The smaller this value is, the better the model is.

(9) $$\mathrm{MSE}=\frac{1}{n}\;\overset{n}{\underset{i=1}{\sum }}{\left({\hat{x}}_{i}-x_{i}\right)}^{2}$$

## 3. The Proposed Models Based on BP Neural Network

In this work, three kinds of pipeline corrosion growth models are constructed and compared. The model 1 is a traditional corrosion model using ANN. The model 2 is a proposed model considering the uncertainties of initial corrosion time and corrosion growth rates. In addition, model 3 is a proposed model which also considers the uncertainties of corrosion length, width, and depth. The traditional corrosion model is set up directly by ANN which is introduced in Section 2. The other two proposed models are introduced in this section.

### 3.1. Data Preprocessing

The data in this paper mainly come from the inspection and evaluation results of major pipelines by Sinopec pipeline storage and Transportation Co., Ltd. from 2015 to 2017. It records the corrosion type, service life, length of pipe segment, distance to upstream girth weld, size and clock direction of the corrosion, and other information, which is shown in Table 2.

Before data processing, some field data, such as the corrosion type and the location of the corrosion, cannot be quantified, so they are classified and numbered before processing. Specifically, we classify four types of corrosion, pit corrosion is recorded as 1, general corrosion is recorded as 2, and the circumferential groove is recorded as 3. The corrosion position is marked as 1 for the inner wall and 2 for the outer wall. In industry, defect direction is denoted by clock, namely hour (*h*) and minute *(m*). In our model, the clock direction is converted to angle according to Equation (10).

(10) $$\theta =720\times \frac{h\times 60+m}{24\times 60}$$

where *θ* is the angle of the corrosion defect. The corrosion depth *d* of the pipeline can also be calculated by using the corrosion percentage *a*% multiplied by wall thickness *t*, as shown in Equation (11).

(11) d=t×a%

After preprocessing the collected field data, we can use these data as input random variables to construct the neural network model.

### 3.2. BP Neural Network Model Considering the Uncertainties of Initial Corrosion Time and Corrosion Depth

Because the pipeline does not begin to corrode immediately after being put into use, but takes time to begin to corrode, it is necessary to consider the uncertainty in initial corrosion time. Here, it is assumed that the initial time $T_{\mathrm{initial}}$ follows a normal distribution when the pipeline begins to corrode. The detail is as follow [25].

(12) $$T_{\mathrm{initial}}∼N\left(T_{0},std_{T_{0}}{}^{2}\right)$$

To construct this proposed ANN model, we first preprocess the input data, which are introduced in last section. Then, we calculate the actual corrosion time *T*_actual_ using Equation (13). To consider the real situation, the corrosion coefficient *O_i_* related to corrosion rate in a short time is obtained according to the established neural network model. The corrosion amount of the pipeline is calculated and accumulated to obtain the corrosion model of the pipeline. Considering the uncertainties of measured value and actual corrosion rate, we assume that the corrosion coefficient follows the normal distribution represented by Equation (14). Then, the corrosion depth can be calculated using Equation (15).

(13) $$T_{\mathrm{actual}}=T-T_{\mathrm{initial}}$$

(14) $${\tilde{O}}_{i}∼N\left(O_{i},2.5\times 10^{-3}O_{i}{}^{2}\right)$$

(15) $$D\left(t\right)=D_{0}+\overset{t}{\underset{i=1}{\sum }}\phi \left({\tilde{O}}_{i},i\right)$$

where the variable *T* is the service time of pipeline; *O_i_* represents the output corrosion coefficient of the *i*th year from the neural network; and *D*_0_ is the initial corrosion depth. The function *ϕ* represents the relationship between corrosion coefficient and corrosion growth rate, so the corrosion coefficient can be taken as the corrosion rate especially for linear growth corrosion. What is more, to reduce the accidental error, the neural network is trained ten times, and the average of training results is used as the prediction result.

### 3.3. BP Neural Network Considering the Uncertainties in Corrosion Size

Due to the limitations in inline inspection tools, there exist measurement errors in detected corrosion size. Hence, it is necessary to consider the uncertainties in corrosion size. In this proposed model, in addition to considering the uncertainties mentioned in model 2, the uncertainties in corrosion size (length, width, depth) are also added to the BP neural network model. The corrosion depth, width, and length can then be calculated by Equations (16)–(18).

(16) $$D\left(t\right)=D_{0}+\overset{t}{\underset{i=1}{\sum }}\phi \left({\tilde{O}}_{D},i\right)$$

(17) $$L\left(t\right)=L_{0}+\overset{t}{\underset{i=1}{\sum }}\phi \left({\tilde{O}}_{L},i\right)$$

(18) $$W\left(t\right)=W_{0}+\overset{t}{\underset{i=1}{\sum }}\phi \left({\tilde{O}}_{W},i\right)$$

where *D*, *L*, and *W* represent the corrosion amount of the pipeline in the direction of depth, length, and width, respectively; and *D*_0_, *L*_0_, and *W*_0_ correspond to the initial corrosion depth, length, and width, respectively.

According to a selected sample, we calculate the corrosion coefficient in three corrosion directions firstly. We assume that there are fixed corrosion coefficients ${\tilde{O}}_{w}$ and ${\tilde{O}}_{l}$ in the width and length directions, respectively. Here, we can calculate the corresponding corrosion amounts of W(t) and L(t). Then, the variations of corrosion amount in these two parameters are included in the input data of the BP neural network, and the corrosion coefficient in the depth direction is the output parameter. By substituting the corrosion coefficient into Equation (16), the corrosion depth can be obtained for further risk analysis. In each simulation run, the variations in the corrosion length and width of the test sample over time are added to the sample data. We use the same input data as training data to obtain this proposed model 3 for future comparisons.

## 4. Case Studies

### 4.1. General Information

In this section, examples are used to demonstrate the effectiveness of the proposed models. The comparison results for the three corrosion models can be used for the subsequent reliability evaluation and risk analysis of pipelines. Table 3 summarizes the differences among three BP neural network models. The pipeline failure caused by a corrosion defect is mainly because the corrosion depth reaches the critical value. So, in the following case studies, we mainly focus on the growth of corrosion depth rather than length and width. Based on the field data, we investigate three types of corrosion depth growth models. In the first case, the depth of corrosion increases with time linearly. In the second case, corrosion growth follows an exponential distribution. As for the third case, the growth of corrosion depth in each period conforms to the gamma growth process.

### 4.2. Case Study 1: Uniform Corrosion Hypothesis

The growth of the defect depth is characterized by:

(19) $$d\left(t\right)=d_{0}+g_{d}t$$

where *d*_0_ represents the initial corrosion amount and *g_d_* is the growth rate of corrosion depth. *g_d_* is used as the output parameter in the neural network model. When considering the uncertainty, we assume that *g_d_* follows the normal distribution, that the actual corrosion depth growth rate conforms to the theoretical value, and that the variance is 0.05 times the theoretical value.

#### 4.2.1. Traditional Linear Corrosion Growth Model (Model 1)

**Figure 2:**
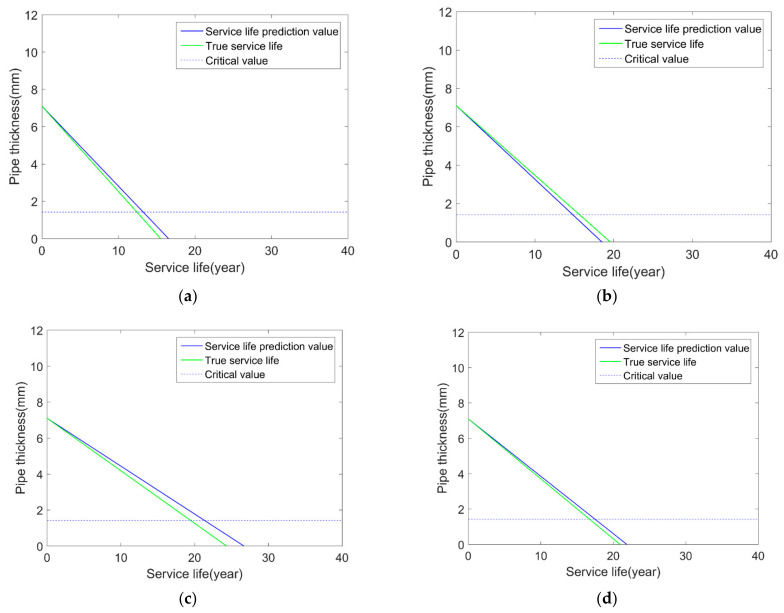
Comparison results of pipeline life prediction: (**a**) sample 1; (**b**) sample 2; (**c**) sample 3; (**d**) sample 4.

The BP neural network is used to simulate the corrosion depth of the pipeline, and the results are shown in Figure 2.

As can be seen from the figure, when the BP neural network method is used to predict pipeline corrosion, the prediction results are promising. The predicted corrosion depth growth rates are relatively close to the theoretical growth rate, which can illustrate the great potential of the BP neural network method in predicting pipeline remaining useful life.

#### 4.2.2. Linear Corrosion Growth Model Considering the Uncertainties of Initial Corrosion Time and Corrosion Depth (Model 2)

**Figure 3:**
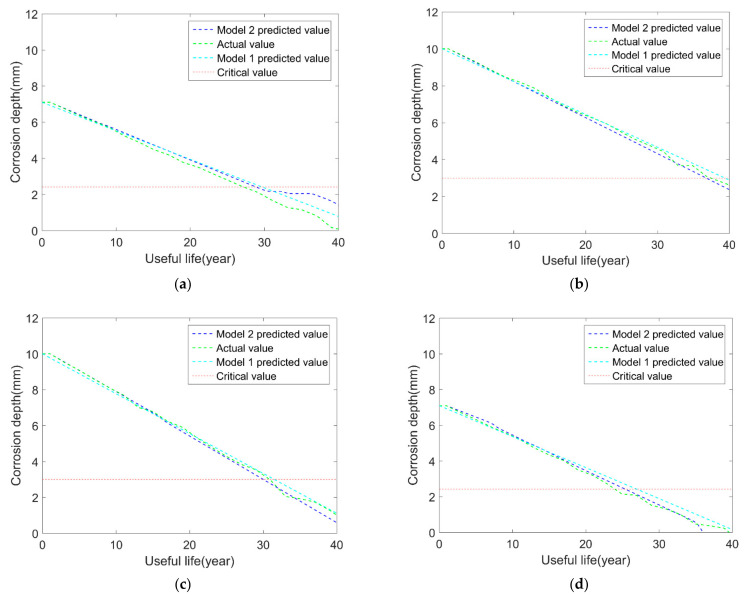
Comparison results of real value, model 1 and model 2: (**a**) sample 1; (**b**) sample 2; (**c**) sample 3; (**d**) sample 4.

With the uncertainties of initial corrosion time and corrosion depth in the model 2, and the prediction results of the model 2 is observed and compared with model 1 in Figure 3. The summary of comparison results is shown in Table 4.

After 20 simulation runs of the corresponding network, the service life of the pipeline is shown in Table 4.

To facilitate the comparison, take the absolute value of error for calculation. It can be obtained that the standard deviation of the error of model 2 is 1.1806, and that the standard deviation of the uniform corrosion of model 1 is 1.7084. Thus, it can be concluded that the neural network model 2 is better than the previous model 1. As can be seen from the above figure and table, the prediction results of model 2 are closer to the real values than model 1, which fully illustrates that the proposed model 2 has better performance of predicting pipeline corrosion growth and remaining useful life.

#### 4.2.3. Linear Corrosion Growth Model Considering the Uncertainties of Corrosion Size (Model 3)

**Figure 4:**
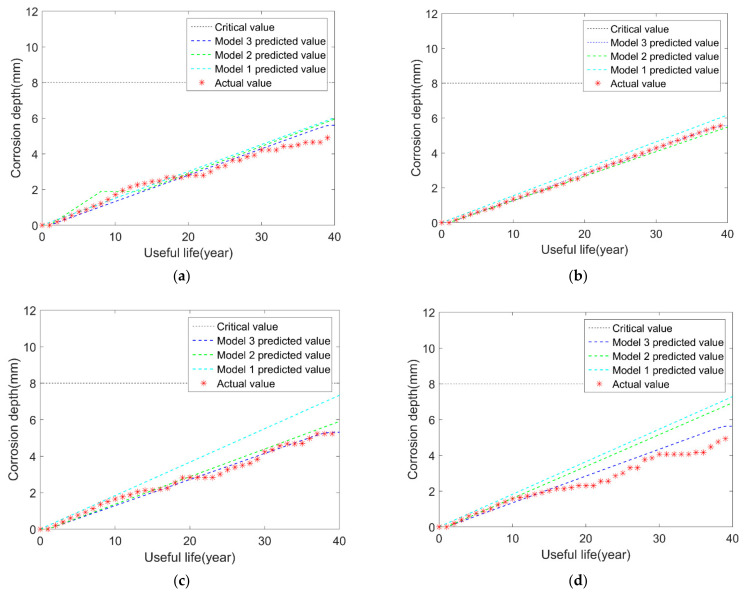
Comparison results of the three models and the real results: (**a**) sample 1; (**b**) sample 2; (**c**) sample 3; (**d**) sample 4.

We build the model 3 based on the previous model 2 and add the uncertainties in corrosion length and width in the BP neural network. The results are shown in Figure 4. Furthermore, to compare the prediction results of the three models more clearly, the mean of squared errors (MSE) between the predicted value and real values is calculated in Table 5.

From the comparison results in the above figures, it can be seen that, after considering the variations in the length and width of corrosion over time, the results of model 3 are closer to the true values compared with model 1 and model 2. At the same time, the above table shows that the MSEs of model 3 are smaller than model 2 and model 1. What is more, the results of model 2 are better than model 1. Considering multiple uncertainty sources, it can be explained that the simulated effects of the above three neural network models are gradually getting closer to reality. Consequently, the proposed BP neural network models produce better results and can make a more accurate pipeline remaining useful life prediction than the traditional BP neural network model (model 1).

### 4.3. Case Study 2: Exponential Model Hypothesis

The three BP neural network models can also be used to predict the exponential corrosion growth model of the pipeline. The growth of the defect depth is characterized by:

(20) $$d\left(t\right)=d_{0}+kt^{\gamma }$$

where *d*_0_ is the initial corrosion depth, and *k* and *γ* are the parameters of the exponential corrosion growth model. Because the variability of *k* is usually large, and the variability of *γ* is usually small [26], the value of *γ* is selected as 0.5. Then, the model parameter *k* is used as the output of neural network models.

#### 4.3.1. Traditional Exponential Growth Model (Model 1)

**Figure 5:**
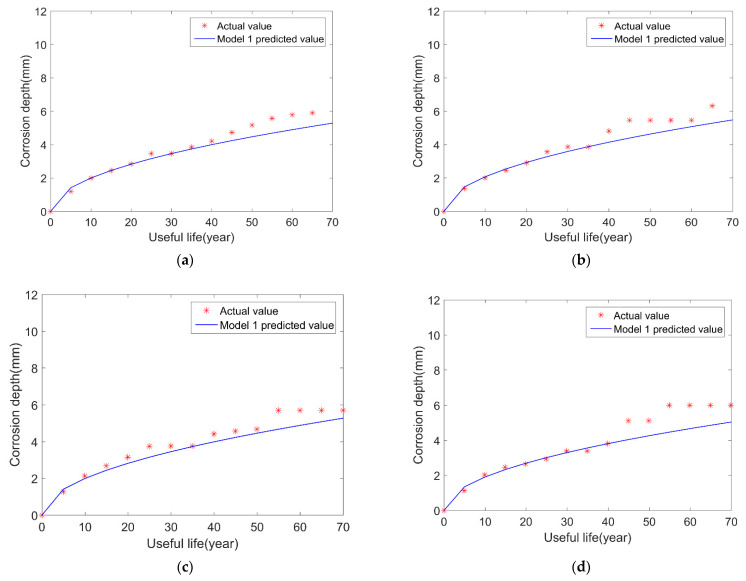
Comparison results of model 1 and the real results: (**a**) sample 1; (**b**) sample 2; (**c**) sample 3; (**d**) sample 4.

No uncertainty is considered in model 1. When constructing the exponential growth model of the pipeline, it is assumed that the time index of the real corrosion rate obeys a normal distribution with a mean value of 0.5 and a variance of 0.05. At the same time, a proportional coefficient is constructed using real corrosion data. We obtain the simulation results every five years. The corresponding results are shown in Figure 5. The simulated results accord with the trend of actual value, but there are big differences as useful life increases.

#### 4.3.2. Exponential Corrosion Growth Model Considering the Uncertainties in Initial Corrosion Time, Corrosion Index, and Scale Factor (Model 2)

**Figure 6:**
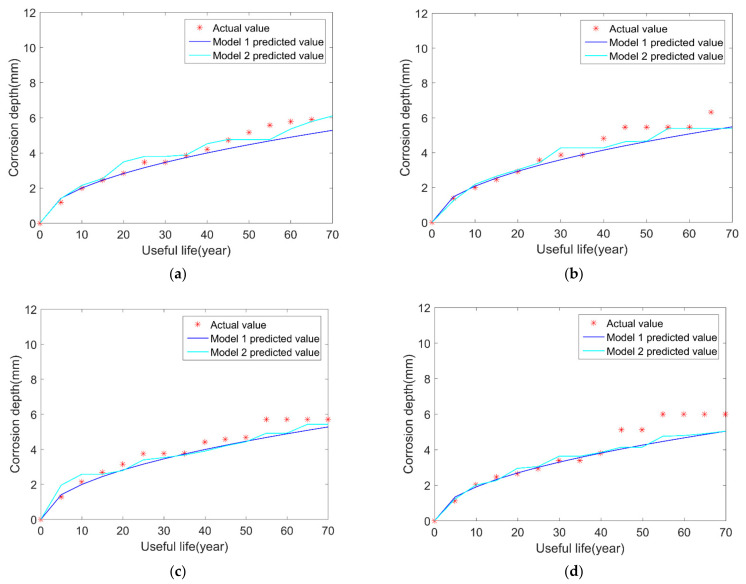
Comparison results of the two models and the real results: (**a**) sample 1; (**b**) sample 2; (**c**) sample 3; (**d**) sample 4.

In the proposed model 2, we consider the uncertainties of the initial corrosion time, the corrosion index, and the scale factor in the BP neural network. The simulation results are evaluated every five years, and the results are compared and analyzed, as shown in Figure 6 and Table 6.

It can be seen from the figures that, among the above four samples, the simulated results of the four figures show that the prediction results generated by model 2 are closer to the true value than model 1. In addition, from Table 6, the standard deviations of model 2 are smaller than model 1, which indicates that model 2 performs better than model 1 in the pipeline corrosion depth growth prediction. However, the prediction results are still not accurate enough for the pipeline remaining useful life prediction.

#### 4.3.3. Exponential Corrosion Growth Model Considering the Uncertainties of Corrosion Size (Model 3)

**Figure 7:**
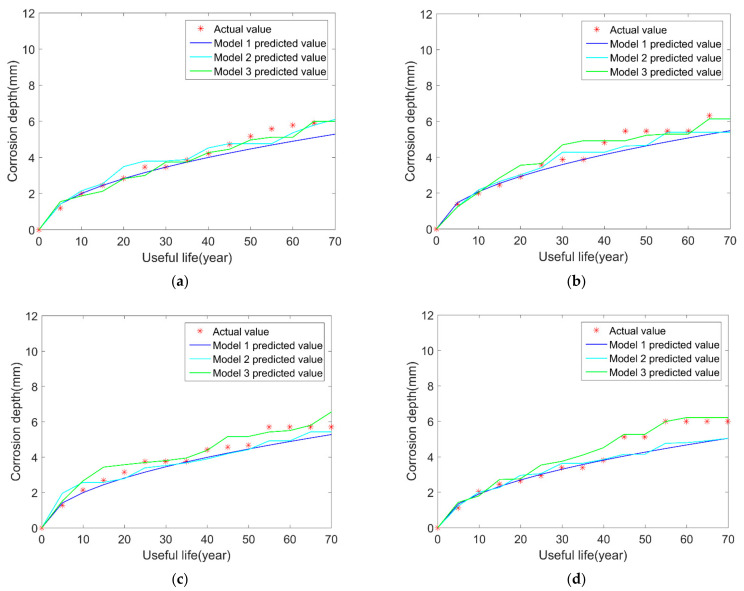
Comparison results of the three models and the real results: (**a**) sample 1; (**b**) sample 2; (**c**) sample 3; (**d**) sample 4.

In addition to considering the uncertainties of initial corrosion time and relevant corrosion depth parameters, the variations in length and width over time are also added to the BP neural network model. Then we compare the results of this new model (model 3) with model 1 and model 2. The results are shown in Figure 7. The differences between the predicted value and real values are also summarized in Table 7.

It also clearly shows that, after considering the changes in the length and width of corrosion over time, the results of model 3 are closer to the true values. In other words, from model 1 to model 3, accuracy and stability are gradually enhanced. Furthermore, compared with model 1 and model 2, the prediction accuracy for model 3 increase a lot, which concludes the uncertainties in corrosion length and width do affect the growth of corrosion depth a lot.

### 4.4. Case Study 3: Gamma Distribution Hypothesis

#### 4.4.1. Traditional Gamma Corrosion Growth Model (Model 1)

**Figure 8:**
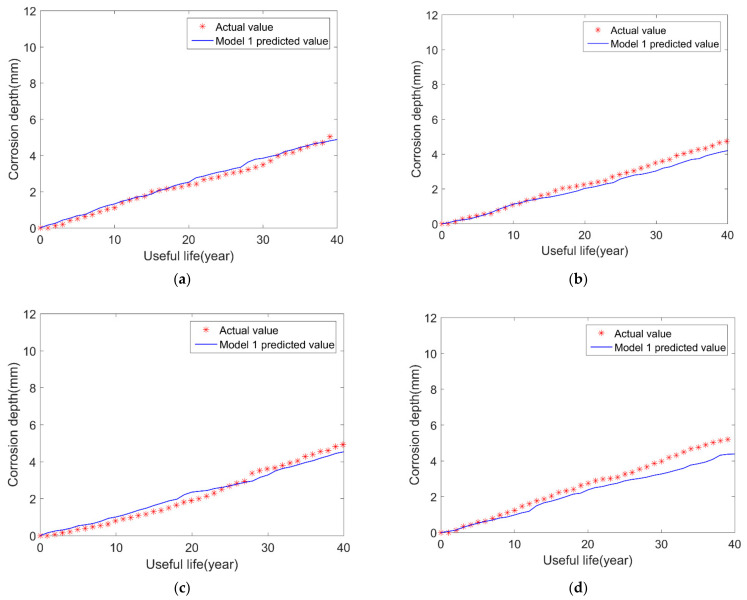
Comparison results of model 1 and the real results: (**a**) sample 1; (**b**) sample 2; (**c**) sample 3; (**d**) sample 4.

In this gamma growth model, it is assumed that the amount of corrosion per year obeys a gamma distribution. The growth of the defect depth is characterized by:

(21) $$d\left(t\right)=d_{0}+d_{g}\left(t\right)$$

(22) $$F(d_{g}\left(t\right)|\alpha ,\beta ,t)=\beta^{\alpha t}\left(d_{g}{)}^{\alpha t-1}\exp(-\beta d_{g}\right)/\Gamma \left(\alpha t\right)$$

where *d_g_*(*t*) denotes the homogeneous gamma process. The probability density function of *d_g_*(*t*) is given by Equation (22), where *α* and *β* are shape and scale parameters of gammar process, respectively. Moreover, in our paper, the scale parameter *β* is assumed to be 48 according to the specific value. We use the shape parameter *α* as the main output parameter of the BP neural network. According to this notion, we establish a BP neural network model, and the results are shown in Figure 8. Furthermore, the prediction results have the same trend with the actual values, but there still are some prediction errors.

#### 4.4.2. Gamma Process Corrosion Growth Model Considering the Uncertainties of Initial Corrosion Time and the Shape Parameters (Model 2)

**Figure 9:**
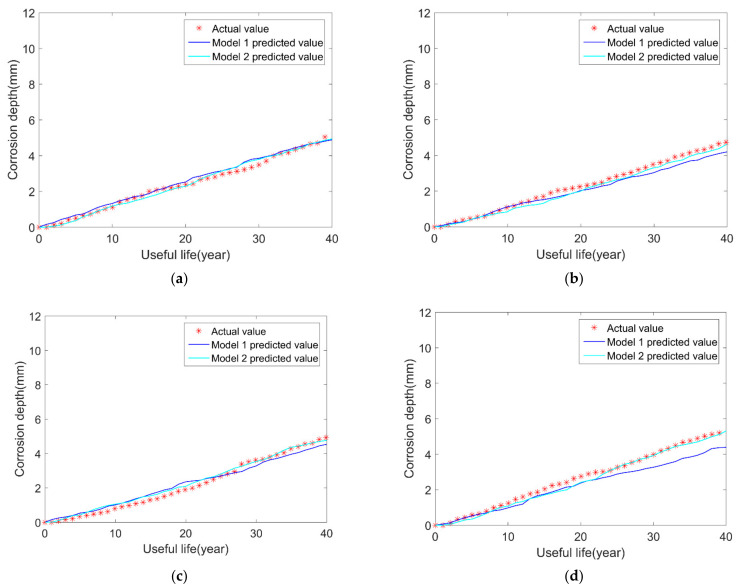
Comparison results of the two models and the real results: (**a**) sample 1; (**b**) sample 2; (**c**) sample 3; (**d**) sample 4.

In this model, the uncertainties of the initial corrosion time and the shape parameters are considered in the BP neural network. We compare this model 2 with model 1 and the actual value. These results are shown in Figure 9.

The tendency of the results is the same as the uniform corrosion model and exponential model. The model which considers uncertainties of initial corrosion time and shape parameters has better prediction results.

#### 4.4.3. Gamma Corrosion Growth Model Considering the Uncertainties of Corrosion Size (Model 3)

**Figure 10:**
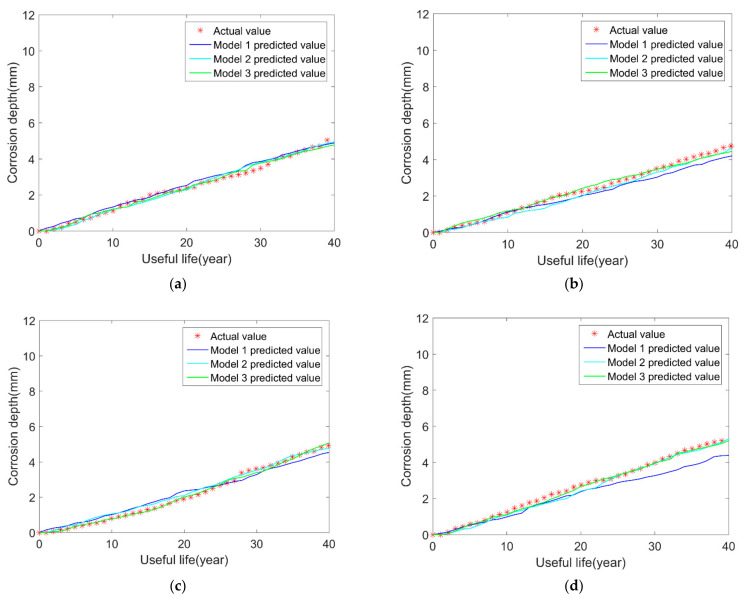
Comparison results of the three models and the real results: (**a**) sample 1; (**b**) sample 2; (**c**) sample 3; (**d**) sample 4.

The steps are the same as linear and exponential growth models, and we also consider the uncertainties in corrosion size over time in the proposed BP neural network. The comparison results for models 1, 2, and 3 are shown in Figure 10. The differences between the predicted value and real values are also summarized in Table 8.

After considering the uncertainties in the length and width of corrosion over time, the results of model 3 are closer to the actual values. Additionally, from model 1 to model 2 to model 3, accuracy and stability are gradually enhanced. As linear, exponential and Gamma corrosion growth models can be used to describe most corroded the pipelines’ degradation working cases. So, we take these three hypothetical growth models as examples, and our proposed BP neural network models’ rationality, effectiveness, and universal applicability are verified.

## 5. Conclusions

This paper proposed BP neural network models for pipeline useful life prediction considering the uncertainties in initial corrosion time and corrosion size. Furthermore, we use the field data from the Sinopec Pipeline Storage and Transportation Co., Ltd. to demonstrate the effectiveness of the proposed model. We first preprocess the collected field data, and we can use these data (pipe parameters, corrosion location, corrosion size, etc.) as input random variables to construct the neural network model. Three gradually improved models are considered in the pipeline RUL prediction, and the uncertainties are added to each model to make the hypothetical pipeline corrosion situation closer to reality. At the same time, by comparing the results of the neural network with the real values, it can be seen that the results are relatively close, which fully illustrates the effectiveness and the rationality of the BP neural network method in predicting the corrosion degree of pipelines. Three proposed BP neural network models are compared with actual values, a corresponding comparative analysis of the results shows that the model 3 which considers the uncertainties from corrosion initial time and corrosion size produce more accurate prediction results. Lastly, we use three case studies to demonstrate the effectiveness of the proposed models. Three types of corrosion growth models, namely uniform, exponential model, and gamma process models, are applied to the proposed models mentioned above. The comparison results prove that the proposed models have universal applicability to different working conditions.

## Figures and Tables

**Table 1:** Parameter selection of back propagation (BP) neural network.

Type	Parameter	Value
1	Number of hidden layer nodes	5,7,9,11
2	Learning rate	0.1
3	Learning objectives	10-7
4	Frequency of training	1000

**Table 2:** Sample of field data.

Sample	1	2	3	4	5
Corrosion type	Circumferential	General	Circumferential	Circumferential	General
Service life (year)	8	8	9	12	18
Length of a pipe segment (m)	12.0	5.7	11.0	11.0	11.9
Distance to upstream girth weld (m)	11.4	2.5	8.3	1.5	11.2
Distance to downstream girth weld (m)	0.6	3.2	2.7	9.5	0.7
Corrosion length (mm)	18.0	80.0	18.0	21.0	30.0
Corrosion width (mm)	87.0	68.0	62.0	75.0	106.0
Clock direction of corrosion	11:00	2:43	6:34	1:54	3:52
Pipe wall thickness (mm)	10.3	10.3	7.1	7.1	8.7
inner or outer	outer	outer	outer	inner	inner
Corrosion percentage	4	7	4	5	4

**Table 3:** Three gradually improved models.

Uncertainty	Model 1	Model 2	Model 3
The initial time of corrosion	no	yes	yes
Corrosion depth	no	yes	yes
Corrosion length and width	no	no	yes

**Table 4:** Analysis of the results of pipeline service life.

No.	Real Service Life	The Outcome of Model 2	The Outcome of Model 1	The Error of Model 2	The Error of Model 1
1	23.9248	25.4248	26.8248	1.5	1.4
2	30.9869	30.5869	32.2869	0.4	1.7
3	20.5586	21.3586	21.9586	0.8	0.6
4	37.4537	36.7537	39.2537	0.7	2.5
5	27.0945	28.8945	29.5945	1.8	0.7
6	25.8102	22.6102	28.6102	3.2	6
7	26.6273	26.0273	28.3273	0.6	2.3
8	35.5698	40.0098	38.0698	4.4302	1.9302
9	37.1356	35.7356	38.4356	1.4	2.7
10	19.6039	18.6039	21.3039	1	2.7
11	32.9597	32.1597	34.0597	0.8	1.9
12	39.095	38.495	40.1	0.6	1.605
13	25.6869	26.6869	26.8869	1	0.2
14	25.0132	26.0132	27.0132	1	1
15	30.8332	30.0332	31.6332	0.8	1.6
16	33.9482	33.0482	35.0482	0.9	2
17	21.4839	20.6389	23.7839	0.845	3.145
18	26.3141	23.1141	29.8141	3.2	6.7
19	21.0338	23.3338	23.6338	2.3	0.3
20	33.499	32.9099	33.1099	0.5891	0.2

**Table 5:** Mean of squared errors (MSE) between the predicted value and real value.

No	The Outcome of Model 1	The Outcome of Model 2	The Outcome of Model 3
1	0.3593	0.2504	0.2026
2	0.2131	0.0846	0.0424
3	0.7162	0.1527	0.1077
4	0.7799	0.6848	0.3004

**Table 6:** Variance of the difference between the predicted value and real value.

	Sample 1	Sample 2	Sample 3	Sample 4
Model 1	0.2963	0.3689	0.2957	0.4914
Model 2	0.2062	0.3416	0.2770	0.4319

**Table 7:** Variance of the difference between the predicted value and real value.

	Sample 1	Sample 2	Sample 3	Sample 4
Model 1	0.2963	0.3689	0.2957	0.4914
Model 2	0.2062	0.3416	0.2770	0.4319
Model 3	0.1664	0.3014	0.2664	0.2429

**Table 8:** Variance of the difference between the predicted value and real value.

	Sample 1	Sample 2	Sample 3	Sample 4
Model 1	0.1120	0.1681	0.1172	0.3019
Model 2	0.1057	0.1121	0.1028	0.1324
Model 3	0.0880	0.0858	0.0687	0.0754

## Data Availability

The data presented in this study are available on request from the corresponding author.
